# Endometriosis, infertility and occupational life: women's plea for recognition

**DOI:** 10.1186/s12905-023-02183-9

**Published:** 2023-01-20

**Authors:** Letizia Gremillet, Antoine Netter, Irène Sari-Minodier, Laura Miquel, Arnaud Lacan, Blandine Courbiere

**Affiliations:** 1 Department of Gynecology-Obstetrics and Reproductive Medicine, AP-HM, La Conception Hospital, 147 Boulevard Baille, Marseille, France; 2 CNRS, IRD, IMBE UMR 7263 FR, AP-HM, Aix Marseille Univ, Avignon University, Marseille, France; 3 Faculty of Medical and Paramedical Sciences, AP-HM La Timone, University Hospital Service of Medicine and Occupational Health, Aix Marseille Univ 27, Boulevard Jean Moulin, 13385 Marseille Cedex 5, France; 4 AMSE, CNRS, UMR 7316, Kedge Business School, EHESS, Marseille, France

**Keywords:** Endometriosis, Qualitative study, In vitro fertilization

## Abstract

The objective of this study was to explore and describe the specificities of the occupational life of infertile endometriotic women treated by in vitro fertilization. We conducted a qualitative monocentric study between December 2020 and June 2021. Twelve semi-structured in-depth interviews using a theme-based interview guide with open questions were undertaken with infertile women with deep infiltrating endometriosis. Data analysis was conducted using an inductive approach according to the grounded theory method. Three main themes emerged from the interviews: (i) barriers to reconciling illness and work life, (ii) facilitating factors for well-being at work, and (iii) consequences and outlooks. It appeared that the time of infertility treatment represents a particular period of change in the working lives of women with endometriosis. For most women, these changes are experienced negatively, often with a renunciation of goals. For others, this is the time to communicate the difficulties linked to their illness to their professional entourage. There is a long path ahead to finally achieving recognition of endometriosis in the context of professional life.

## Introduction

Endometriosis is a chronic disease that affects approximately 10% of women of childbearing age [1]. This disease is defined by the presence of ectopic endometrial tissue (i.e., appearing outside the uterine cavity), most often on the ovaries or the peritoneal cavity, but sometimes infiltrating the tissues in the form of deep pelvic endometriosis [2]. The main symptoms of this disease are intense dysmenorrhea, dyspareunia and chronic pelvic pain. Women with endometriosis also often have difficulty conceiving and often have to resort to assisted reproduction or surgery to achieve pregnancy [3, 4]. These elements are known to have a negative impact on the intimate, social and professional lives of patients [5].

Endometriosis affects women during their reproductive years, with an average delay in diagnosis of 7 years; thus, the diagnosis of the disease is most often made at a time that coincides with the beginning of a woman’s professional life [6, 7]. Although the economic impact has been well studied quantitatively by cross-sectional studies, the long-term consequences on professional development and the impact on the career path are still unknown. Several qualitative studies have been conducted on the subject of endometriosis and have provided a better understanding of its impact on general quality of life, social relationships and intimate life [8, 9]. Recently, Courbiere et al. showed that infertility and assisted reproduction had a significant impact on quality of life at work, with 49% of people undergoing treatment with assisted reproductive technology (ART) reporting negative repercussions on the quality of their work [10]. To date, no qualitative study has been conducted to investigate the specific matter of the professional impact of endometriosis in the context of infertility.

The objective of this study was to explore and describe, through a qualitative study with semi-directed interviews, the specificities of the occupational life of infertile endometriotic women treated by in vitro fertilization.

## Materials and methods

### Type of study

We conducted semi-structured in-depth interviews using an interview guide with open questions. In order to obtain qualitative data on the experience of infertility in the context of deep infiltrating endometriosis, the questions were mainly about the experience and understanding of the disease in the professional sphere: the way in which the pain symptoms and infertility are perceived by work colleagues, subordinates and superiors; the possible hindrance to hiring; the repercussions on the quality of work; the justification for absences (absenteeism); and the loss of productivity (in connection with presenteeism). Among unemployed women, we also studied the extent to which their pathology could be an obstacle to returning to work.

### Study population

The study participants were recruited during gynecological follow-up consultations for infertile women with endometriosis at La Conception Hospital ART center in Marseille, France.

The study included women aged 18–43 undergoing gynecological follow-up for infertility in the context of documented severe endometriosis. Stage IV (severe endometriosis) of the rASRM classification had to be suspected (on MRI) or proved (by surgery). Women with an associated chronic pathology or poor understanding of the French language were not included. Women could be included if they had a paid employment or if they were self-employed. In order to also take into account women who might have lost their job due to the disease, it was decided to include unemployed women provided that they had already had a paid job in the past. All participants provided informed consent by signing a written consent form.

The study was approved by the local ethics committee of Aix-Marseille University (2021-01-07=07). All methods were carried out in accordance with relevant guidelines and regulations.

### Semi-structured in-depth interviews

Individual, in-depth, semi-structured interviews were conducted exclusively by telephone in accordance with the health protocols in place due to the COVID-19 pandemic by a single investigator (L.G.). Signed consent was requested from participants before the interview began. The expected duration of the interviews announced to the participants was 30 min to one hour. A semi-structured interview guide composed of open-ended questions concerning the experience and understanding of the disease in the professional sphere enabled the investigator to conduct each conversation in a semi-structured manner (the interviewer has a list of topics and questions to ask yet these questions are not too specific so as to allow a wide variety of answers). The women were encouraged to develop their experiences and ideas in a detailed and nuanced way (in-depth) by being prompted by new free-form questions chosen by the interviewer. Each interview was recorded and immediately transcribed *verbatim*, removing any information that might identify the participant. The recording was then deleted.

### Data collection and analysis

Data analysis was conducted using an inductive approach based on the grounded theory method, which aims to generate theories from empirical material [11].

The data were anonymized and then manually analyzed by coding. The aim of the analysis is to resolve the complexity of the large number of in-depth interviews and thus to report on the data in a comprehensive way yet classified into thematic categories. This should allow the details and nuances of the participants' responses to be set out in an understandable way so that assumptions can be made about the impact of endometriosis on working life. The first step was to read the verbatim transcripts of each interview and classify each sentence/idea into different categories. The second step was to group the categories into themes. The third step was to assemble these themes into more general concepts [12, 13]. The data were analyzed progressively along with the interviews and the themes and categories were adjusted according to the data collected, following the principle of constant comparative analysis [14–16]. The individual interview guide was refined over the course of the study in light of the emerging results. The literature search was conducted at the end of the study to ensure that the analysis of the data remained neutral and was not influenced by themes presented in other studies.

## Results

### Characteristics of the population

Interviews were conducted with 12 women and lasted 28–65 min. The mean age was 33 ± 3.3 years (range 29–40 years). The mean age of onset of symptoms was 15 ± 2.8 years (range 13–17 years), and the mean age at diagnosis was 29 ± 2.6 years (range 23–32 years). Five patients had undergone surgery because of their endometriotic pathology. Their professional status is listed in Table 1.

**Table 1:** Women’s characteristics

Participants	Age (years)	Age at diagnosis (years)	Surgery for endometriosis	Parity	Time since the beginning of IVF (Months)	Occupational status
P1	35	26	/	0	6	Public employee
P2	33	31	/	0	24	Unemployed
P3	35	25	/	2	36	Public employee
P4	38	32	/	0	36	Merchant
P5	30	23	Yes	0	24	Employee
P6	31	27	Yes	0	6	Employee
P7	40	28	Yes	1	24	Artisan
P8	38	25	Yes	0	6	Artisan
P9	35	26	/	0	6	Merchant
P10	31	26	/	0	12	Merchant
P11	33	30	Yes	0	48	Public employee
P12	29	26	/	0	6	Employee

*IVF* In Vitro Fertilization

Three main themes emerged from the interviews: (i) barriers to reconciling illness and work life, (ii) factors facilitating well-being at work, and (iii) consequences and prospects.

### Obstacles to balancing illness and work life

#### A physical hindrance, a psychological impact

Cyclic pelvic pain is one of the most frequent clinical manifestations of endometriosis and is the first symptom reported by patients as representing a handicap when it occurs during their professional activity.

“It’s difficult to pretend that everything is fine in front of clients when the pain comes like a stab wound.” P4

This fear leads patients to anticipate periods of crisis and to find ways to cope with them.

“I anticipate, I’m really afraid of these digestive crises because I’m unable to do anything, I'm paralyzed by the pain. So I anticipate, sometimes I take days off during my period.” P5.

“I would sit with a hot water bottle in my pants, I was still present at work.” P11.

The working conditions were not the same for all patients. Some managed to work in analgesic positions, sitting most of the time, while others did not even have access to a lavatory during their working hours:

“I was having a very difficult time at work, as I spend entire days in my car. I had a very painful episode behind the wheel recently with blood clot loss, soiling all of my clothes and car seat because I had no place to stop.” P6.

“I am standing up all day and am not allowed to go to the bathroom as frequently as I should.” P7

In addition to the painful symptoms of endometriosis, the management of infertility was also perceived as a hindrance to productivity, both because of the side effects of hormonal treatments and because of the stress linked to the multitude of medical examinations and procedures.

“What impacts my life in general is the IVF because my hormone treatment causes me nausea, dizziness, I feel nervous and often exhausted.” P10

“Sometimes I am slower, less focused and have a harder time doing my tasks properly, especially when I am waiting for my blood test results.” P12

#### A shameful disease

More than half of the women interviewed admitted to keeping silent about their disease in the workplace out of shame, fear of judgment or the feeling of weakness it engendered.

“I felt like I was being judged, like I wasn’t diligent enough or helping my colleagues enough. I came across as a girl who wasn’t serious with her stomach pains.” P1

“It's the fear of being judged. I didn’t want people to think I was fragile, that I was going to get sick or take more sick days than others.” P6

“I wanted to prove that I was capable of working as hard as someone who is not sick.” P3

Several patients reported that they would rather lie to justify repeated absences. These patients believed that the pain related to endometriosis would not have been perceived by their professional environment as a serious enough reason for absences. Some patients also suggested a link between this lack of understanding and the predominantly male environment in which they worked. Finally, some patients described having no choice but to hide their illness in fear of losing their position or their job:

“I even had to lie and say I had locked my back to stay home, I was in so much pain, but if I said it was my period I wouldn’t be taken seriously.” P4

“It was very difficult to talk about, especially since I only work with men. They don’t really understand and are completely disconnected from that reality. It was very complicated.” P9.

“In the retail industry, we are all pawns. If we start to have health concerns, we are offered another lower position.” P2

#### Dealing with absenteeism

Most of the women interviewed said that although the pain regularly made work difficult or impossible, they always found a way to avoid taking sick days. Often the women reported that they believed this would be ‘frowned upon’ by their work environment, and several also did not recognize the legitimate need for medical leave.

“I try not to take time off work, because I don’t want it to fall on my co-workers.” P3

“I prefer to get organized and schedule days off or time off in lieu during my period.” P8

However, this already precarious approach becomes extremely challenging when beginning in-vitro fertilization (IVF) treatment. Due to the increase in the number of medical appointments and the significant effects of the treatment, some patients who were used to managing their schedule according to their pain quickly find themselves overwhelmed. Several patients described feeling like they were holding down two jobs with conflicting schedules:

“One of the most difficult things to arrange when undergoing ART treatments is balancing work with clinic appointments.” P10

“The days I must go to the fertility clinic are challenging. I go there very early and rush to get to work on time.” P8

Other women chose to put their professional projects on hold to focus solely on their pregnancy project:

“Right now, with the IVF, I don’t really have time to devote to looking for a new job, because I’m focused on my desire to get pregnant.” P2

“I decided to start treatment after my fixed-term contract because I don’t want to have to justify being absent from a new job.” P9

### Factors facilitating well-being at work

#### A liberated word

Most patients stated that they were unwilling to talk about endometriosis, pain and fertility treatment in their professional environment. However, the few patients who chose (or had the opportunity) to openly discuss these matters with their employers and colleagues consistently described a great improvement in the quality of their work:

“I felt relieved, I was worried about it and finally: no discrimination, no judgment.” P8

However, these women admitted that they would never have been able to take this step in the absence of a steady professional position. Several women described that the combination of a secure permanent position and the start of IVF treatment were the main factors that motivated communication in their professional environment. It also appears that the IVF process and infertility are perceived as a more concrete subject and easier to talk about in the professional environment than the painful symptoms of endometriosis.

“I talked about it after a year, at the individual interviews that are done regularly. It was a good time to tell them about my symptoms and the in-vitro fertilization that was being scheduled.” P1.

“I have been in the IVF program for three years now and it is easier for me to justify my absences. […] I feel more legitimate in the context of my pregnancy project than in the context of simple stomach aches. I feel like my boss and colleagues understand my situation better.” P10.

Many women admitted that they had specifically chosen a female interlocutor from their managerial hierarchy to speak up on their behalf. They often felt that a woman would be better able to understand their situation and respond in an empathetic way.

“I went through my direct HR manager, a woman. It was easier that way. She made the connection with the rest of my colleagues, she explained to them that I was undergoing an intervention, that I needed time, a few days a month, without specific details, but she was the one who made the gesture to make them understand my situation.” P3.

Finally, some patients were able to express themselves by referring directly to French law, which allows them to be absent from work in the case of reproductive treatments.

“For this second job, I was able to communicate the legal texts to justify my absences. Everything went well, they accepted my requirements.” P4

#### Organizational flexibility: a determining factor

Patients unanimously described the organizational complexity of coordinating the professional schedule with the scheduling of IVF. It appears that the experience of this period is better for women whose jobs are more flexible in nature and whose colleagues and superiors are more accommodating.

“I’m lucky because whenever I’m not doing well, my colleagues support me. If I need a break, I know I can count on them.” P4

“My working hours were not fixed; I could move my classes around. By playing on all the parameters, I was able to keep working. I've hardly ever been off work.” P11

“Being self-employed, it’s easier to get organized, I don’t work on the days I have medical appointments.” P7

The COVID-19 pandemic and the advent of remote work was also described by many patients as a lull in all organizational difficulties.

“The health crisis we are going through is a blessing in disguise. Remote work allows us to remain efficient but be able to manage our pain.” P1

### Consequences and outlooks

#### A career path shaped by the disease

More than half of the participants felt that endometriosis had a negative impact on their career. This sometimes meant a voluntary or encouraged career change, dismissal or simply a slower progression than their colleagues.

“I wanted to be a police officer, but there are a lot of sports activities and it’s totally impossible for me during my periods. I had to give up this job.” P2

“I am making a professional change with regrets. I liked the job I was doing; I could have moved up the ladder. But physically I could no longer hold on. I had to make a compromise.” P8.

Many participants blame the infertility journey that forced them to make the choice between having children and continuing their career path.

“The desire to have a child is stronger than my career. I gave up my application for a management position.” P12

“For the first IVF, I had to quit my job because I felt judged by my employers and colleagues.” P10

#### A request for recognition

When asked what could have improved their experience, patients said that more recognition was needed in the workplace. According to them, this recognition should first and foremost involve better overall information on endometriosis for the public in general as well as for professional circles. Measures to adjust working hours according to symptom periods and to increase flexibility of timing and location of work were also frequently mentioned. Many women were also particularly informed about these types of measures already in place in other countries.

“We need to talk about it in companies and in schools, so that people will be more understanding and react positively.” P5

“Some countries have adopted a day of rest per week when you have your period, this is a great progress. I don't understand why endometriosis is not recognized as an illness. I would like to have an adjustment of working hours, or remote working.” P2.

According to the participants, two different actors must participate in these steps of recognition at work: the reproduction physician should systematically inform the patient of her professional rights during the treatment period, and the occupational physician should support the adaptation of the position or schedule or facilitate remote work.

“I had one medical visit at occupational medicine, but he probably didn’t know about the disease either. I wish he would have coached me, talked to management to make things easier.” P6.

“The reproductive doctor that follows me always asks me if everything is going well at work. Besides, she’s the one who showed me the legal texts for my repeated absences.” P5

## Discussion

To the best of our knowledge, our study is the first qualitative study investigating the professional impact of severe endometriosis in infertile women. Previous qualitative studies have focused on the quality of life of patients with endometriosis, which is often affected by pain, the emotional impact of infertility, anger about the recurrence of the disease, and uncertainty about the future regarding repeated operations or long-term medical therapy [17]. From the patient’s perspective, endometriosis can be a nightmare of misinformation, myths, taboos, lack of diagnosis, and problematic and uncertain treatments, coupled with a painful, chronic and recalcitrant disease [18]. The impact includes fertility, sexuality, ability to work and personal relationships. Numerous quantitative studies have also investigated the impact of endometriosis on quality of life and the value of different medical and surgical interventions to improve it [19]. Several authors have already investigated the occupational impact of endometriosis through studies mainly based on quantitative questionnaires. The prospective study by Simoens et al. conducted in 10 different countries on 909 women with endometriosis estimated that the average cost per woman in terms of lost productivity was €9579 per year, similar to the average cost of health care expenditure for other chronic diseases such as ankylosing spondylitis and rheumatoid arthritis [20].

In our study, we chose to focus on infertile women because we hypothesized that the use of ART treatments had a significant impact on the professional sphere, both on the respondents’ individual professional lives and on the corporate environment. Indeed, there is a continuum for patients with endometriosis, particularly regarding absenteeism, which is often present from the beginning of working life, especially during menstruations, and which increases during ART treatments. Thus, the beginning of IVF is a pivotal period which often marks a professional rethink. Our qualitative study allowed us to highlight the obstacles to overcome and to better understand the strategies that women use to build their career paths. This study not only allowed us to identify the surface problems that make work difficult for patients with endometriosis (pelvic pain, absenteeism related to infertility treatment) but also to highlight the foundations of the professional world that are aggravating factors for these patients (lack of recognition of endometriosis, difficulty in talking about a health problem affecting women in a typically male professional environment).

The most frequently reported symptom among the women interviewed was pain, which sometimes made it impossible to work. Digestive and urinary problems are often associated with this, as well as very heavy metrorrhagia, forcing women to stop working several times a day. These constraints have also been described in several quantitative studies, which have found an average reduction of 6.3–10.8 h per week of effective work for women with endometriosis compared with a control population [5, 21]. The cyclical and sometimes unpredictable nature of these disorders requires anticipation and vigilance, which can lead to difficulty in concentrating, chronic fatigue, anxiety and irritability, to which can be added the impact of ART treatments. A previous study by our team reported on the psychosocial burden of ART treatments, which can lead to depressive symptoms, absenteeism and professional instability [10]. These challenges are not always disclosed to employers and colleagues. The reasons for non-disclosure are numerous. One reason is the fear of judgment and negative reactions from the professional environment that may lead to repercussions. Another is related to the gender-specific nature of endometriosis, as some women are not willing to discuss their symptoms with male employers, as also reported by Jones et al. [22]. The duality between the preservation of privacy and thorough explanations is consistent in women’s speech. Often, women speaking up about their illness is motivated by three main goals: preserving good organization at work, promoting better information, and protecting oneself from blame [23]. The announcement is often made in the same way: during an off-the-record meeting with their superior. Women also felt that it was more legitimate to justify their absences by the medicalization of their infertility. At no time do the women have to talk about their intimate experiences, but only about a protocol punctuated by examinations that must respect a strict schedule. While some women go to great lengths to arrange their medical appointments and work schedules so as not to inconvenience their colleagues, fewer women choose this time to talk about their illness to their professional entourage. The latter generally describe this period as a turning point in their professional life, often with a good understanding and support from their professional environment, and with work made easier as a result.

The IVF process is punctuated by appointments and multiple examinations that are difficult to anticipate, thus requiring availability and organizational flexibility. There is likely to be an inequality in women’s ability to cope with work-related challenges depending on the nature of their employment. Thus, it appears to be easier for women with flexible schedules and organized telecommuting days or for self-employed women to reconcile these two imperatives. Other women were able to turn to their doctors to obtain sick leave or to assert their right to be absent for infertility treatments according to the French law [24]. Nevertheless, more than half of the women interviewed in our study reported having to give up professional projects. For some, the involvement in their pregnancy project made them give up on obtaining a position of responsibility. These findings are similar to those found in the literature, particularly the cross-sectional study by Fourquet et al. [25] involving 193 women with endometriosis: 40% of the respondents believed that their career was directly and negatively affected by the consequences of endometriosis, in particular because of absenteeism, poor performance, failure to achieve promotion, failure to obtain bonuses, missed professional seminars and loss of clients [8, 26–28]. Finally, many regret the lack of information and recognition of the disease in the workplace. While information can lead to greater freedom of expression, recognition of the disease ensures a feeling of legitimacy and collective management of the career path, particularly with the help of occupational medicine.

The qualitative nature of the study is a strength as it allows for the analysis of specific and nuanced new evidence directly from the patients' experience. Nevertheless, it can also be considered as the main limitation to meet the objective of this study. Thus, the absence of quantitative data does not allow for a definitive conclusion and the interpretation of the interviews only allows for the formulation of hypotheses that must be validated by other studies. Other aspects could also have been studied, such as the impact of the type of endometriosis, surgery or IVF on the quality of life of patients in their work. Quantitative data on the positive or negative impact of the COVID 19 pandemic, which led in particular to an increase in teleworking, would also be interesting. Also in this context, the fact that the interviews were conducted by telephone could have limited the perception of non-verbal elements and diminished the quality of the interaction between the interviewer and the patients. Furthermore, although our study focused on the impact of endometriosis symptoms, infertility and IVF on women's occupational life, other aspects should be addressed such as the impact of surgery (with or without complications) and pregnancy. These have already been studied in relation to quality of life but are likely to have an impact on working life as well [29–32].

## Conclusion

The results of our study suggest that the time of infertility treatment represents a particular period of change in the working lives of women with endometriosis. For most women, these changes are experienced negatively, often with a renunciation of goals. For others, this is the time to communicate the difficulties linked to their illness to their professional entourage. It appears that there is a long path ahead to finally achieving recognition of endometriosis in the context of professional life.

## Data Availability

The dataset generated during and analysed during the current study are available in the Figshare repository, https://dx.doi.org/10.6084/m9.figshare.21436176.
